# Can Appropriate Thermal Post-Treatment Make Defect Content in as-Built Electron Beam Additively Manufactured Alloy 718 Irrelevant?

**DOI:** 10.3390/ma13030536

**Published:** 2020-01-23

**Authors:** Sneha Goel, Kévin Bourreau, Jonas Olsson, Uta Klement, Shrikant Joshi

**Affiliations:** 1 Department of Engineering Science, University West, Trollhättan 46186, Swedenshrikant.joshi@hv.se (S.J.); 2 Specialty Materials, University of Limoges, Limoges 87000, France; 3 Department of Industrial and Materials Science, Chalmers University of Technology, Gothenburg 41296, Sweden

**Keywords:** additive manufacturing, electron beam melting, defects, microstructure, hardness, alloy 718, hot isostatic pressing, post-treatment

## Abstract

Electron beam melting (EBM) is gaining rapid popularity for production of complex customized parts. For strategic applications involving materials like superalloys (e.g., Alloy 718), post-treatments including hot isostatic pressing (HIPing) to eliminate defects, and solutionizing and aging to achieve the desired phase constitution are often practiced. The present study specifically explores the ability of the combination of the above post-treatments to render the as-built defect content in EBM Alloy 718 irrelevant. Results show that HIPing can reduce defect content from as high as 17% in as-built samples (intentionally generated employing increased processing speeds in this illustrative proof-of-concept study) to <0.3%, with the small amount of remnant defects being mainly associated with oxide inclusions. The subsequent solution and aging treatments are also found to yield virtually identical phase distribution and hardness values in samples with vastly varying as-built defect contents. This can have considerable implications in contributing to minimizing elaborate process optimization efforts as well as slightly enhancing production speeds to promote industrialization of EBM for applications that demand the above post-treatments.

## 1. Introduction

Electron beam melting (EBM) is a powder bed fusion-based metal additive manufacturing (AM) technique bearing capability to produce components with high design flexibility. The material systems which can significantly benefit from EBM technology include high-performance materials such as Ni-based superalloys, e.g., Alloy 718, and Ti-alloys [1,2]. In the beginning, a vast majority of the research activity relating to EBM was focused on Ti and its alloys, particularly Ti-6Al-4V [3,4]. Since incorporation of defects (typically gas and shrinkage porosity, and lack of fusion) is a concern during EBM production, there have been several reported efforts with emphasis on process understanding and optimization to curtail defect formation in EBM manufactured Ti-6Al-4V [5,6,7,8]. Moreover, use of unoptimized process parameters has been shown to result in extensive defect formation [8].

Such defect formation in EBM Alloy 718 can also have particularly adverse consequences, as defects can degrade mechanical behavior of the EBM-built material. For instance, anisotropy in tensile behavior of heat-treated EBM Alloy 718 has been attributed to remnant defects [9,10]. The formation of the above defects is not only attributable to the use of unoptimized process parameters but can also occur due to stochastic instabilities in the process. These can include a temporary decrease in electron beam power, non-uniform spread of powder, poor sintering of the powder prior to melting, spattering of powder from the build layer due to excessive electrostatic forces in an event called “smoking”, etc. [11,12,13]. All of these have been reported to cause formation of lack of fusion defects [13]. Consequently, a set of post-treatments are typically considered to enhance the properties of the as-built material [14,15,16] and are deemed particularly necessary in the case of the demanding applications that Alloy 718 is routinely employed for.

In this context, even with use of optimized parameters, defects, to some degree, are inherent to EBM processed Alloy 718 [17]. Consequently, the as-built material is subjected to hot isostatic pressing (HIPing) to close the defects present in the material [18]. In addition, the HIP’ed material is typically subjected to solution treatment and aging to achieve the required phase composition. In this context, it is pertinent to mention that the typical recommended parameters for HIPing are 1120–1185 °C, 100 MPa, 4 h, to be followed by a 1 h solutionizing treatment and a two-step aging involving a 8 h treatment for each step, which is perhaps “borrowed” from what has been the practice with wrought Alloy 718 [19]. Therefore, a holistic approach of optimizing the process, together with applied post-treatments, is relevant from a technology industrialization standpoint. In this context, prior work in the authors’ group has shown that the aging treatment can potentially be shortened considerably to (4 + 1) h instead of (8 + 8) h [20]. Reduction in processing time and costs are also pivotal to industrial exploitation of the EBM technology. Sames et al. [18] have reported that a major limitation is the processing speed. However, faster production speeds are known to lead to poor surface finish and/or increased defect content [18,21]. Specifically, in cases where such post-treatment involving HIPing is deemed inevitable, there could be an opportunity to increase the EBM processing speed without being constrained by the need to eliminate or minimize defects.

The present study explores the potential of the combined post-treatments involving HIPing, solutionizing and aging treatment to test the hypothesis of whether they can render the as-built defect content in EBM Alloy 718 irrelevant. The defect content in the samples was intentionally tailored by systematically increasing the hatch line spacing (LS), which refers to the spacing between raster scanning lines during EBM processing. The extent of defects thus incorporated far exceeded those typically encountered in EBM builds for the sake of this illustrative proof-of-concept study.

## 2. Experimental Procedure

**Figure 1:**
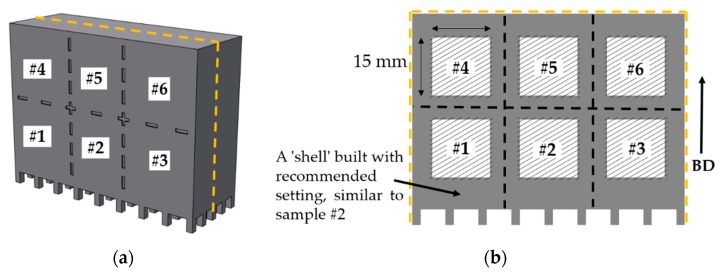
Schematic of the CAD model of one of multiple identical blocks comprising the electron beam melting (EBM) build (**a**), and cross-section view of the block revealing the cubes (marked by hatched lines) processed with different line spacing (**b**). The arrow on the right shows the build direction, BD.

Samples were EBM built with different defect contents by varying LS to assess their response to identical post-treatments, particularly HIPing. Plasma atomized Alloy 718 powder (AP&C, Québec, Canada) [5] recycled ~20 times was used. Six different samples (15 × 15 × 15 mm^3^) (Figure 1), henceforth referred to as AB#1 to AB#6, were built using EBM (Model A2X, Arcam AB, Gothenburg, Sweden) with varying LS corresponding to different processing speeds (expressed as relative melting time and relative melted area per unit time by normalizing with the “standard” values), as summarized in Table 1. All the samples were melted by a snake hatch method during which the electron beam was moved in a back-and-forth raster scan pattern [18]. All the other process parameters were kept constant as per the EBM process theme version 4.2.205, and some of the key process parameters associated with this theme are listed in Table 2. It is worth mentioning that the parameter speed function dynamically adjusts the electron beam scan speed depending on the scan length [22].

Selected samples were further subjected to two different HIPing treatments (HIP1, HIP2). The choice of parameters for HIP1 (1120 °C/100 MPa/4 h/rapid cooling) and HIP2 (1185 °C/100 MPa/4 h/rapid cooling) was based on recommendations of the ASTM (F3055) standard for post-treatment of powder bed fusion-produced Alloy 718 [19]. For one of the HIPing conditions selected based on ensuing results, the samples were also subjected to solution treatment (954 °C/1 h/water cooling), and double aging (740 °C/4 h, cool at 55 °C/h to 635 °C, held at 635 °C/1 h/air cooling) called HIP1 + STA. Details of the above mentioned post-treatments are summarized in Table 3. It is relevant to mention here that the aging protocol reflected above corresponds to a shortened heat treatment compared to the schedule recommended in the above ASTM standard and is based on prior work carried out in this group [20].

**Figure 2:**
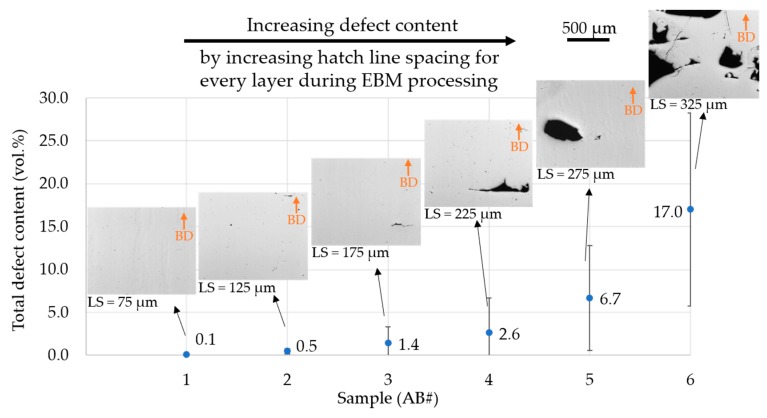
Defect content in as-built samples intentionally built with varied hatch line spacing (LS). The size and amount of defects are visualized in the insets. Arrows in the inset indicate build direction.

All the investigated samples were sectioned along the build direction using an alumina cutting blade. The sample cross-sections, each 15 × 15 mm^2^, were hot mounted, ground, and polished. Microstructural investigation of the prepared samples was carried out using an Olympus BX60 M optical microscope (OM), and HITACHI TM3000 (equipped with energy-dispersive X-ray spectroscopy (EDS)) and LEO 1550 Gemini scanning electron microscopes (SEMs). Quantification of defects was done using OM micrographs of unetched polished samples processed using ImageJ software. In each case, more than 10 images were analyzed to get a representative value of the defect content (magnification: 50×, analyzed area: ~21 mm^2^). For characterization of secondary phases using SEM imaging, the selected samples were electrolytically etched using 50% diluted (in ethanol) Kalling’s 2 reagent (2–3 V applied for 3–5 s). Vickers micro-hardness testing (HMV-2, Shimadzu Corp., Japan) on selected samples was performed using a 500 g load which was applied for 15 s in ambient conditions. Up to 25 random indents were made on each of the samples. Since some of the defect sizes in the intentionally prepared high-defect-content builds were very large (as shown in Figure 2) and exceeded the indent size, few extreme outliers exhibiting “zero” hardness were noted whenever the indent happened to coincide with or be in the immediate vicinity of a very large defect. Average hardness values (excluding only such extreme outliers) are reported herein.

## 3. Results and Discussion

Since the main theme of the paper was to assess whether defects in the as-built condition matter if the subsequent post-treatment steps involve HIPing in any case, results pertaining to as-built conditions are presented first, followed by their response to post-treatments.

The as-built microstructures of samples processed with varying LS corresponding to different melting speeds (Table 1) are depicted in Figure 2. Increase in LS above the “recommended” value resulted in a rise in the defect content, as shown in Figure 2. The defect contents stated represent the sum of all types of defects observed in the as-built samples, i.e., gas and shrinkage porosity, and lack of fusion. Incidentally, these defects were also present in samples produced at the recommended LS = 125 µm (AB#2) as well as at lower LS = 75 µm (AB#1). Samples intentionally produced with higher LS exhibited defect contents up to 17%, as shown in Figure 2 and also visualized in accompanying micrographs. In a word, a systematic increase in line offset (processing speed) from 75 to 325 µm resulted in an increased defect content ranging from 0.1% to 17% in the investigated samples. It is worth mentioning that large defects such as those observed in samples AB#5 (LS = 275 µm) and AB#6 (LS = 325 µm) are not typical of an EBM built even with not fully optimized processing conditions [23]. However, these samples were produced for the present ‘proof of concept’ investigation regarding the extent of defect closure enabled through HIPing.

**Figure 3:**
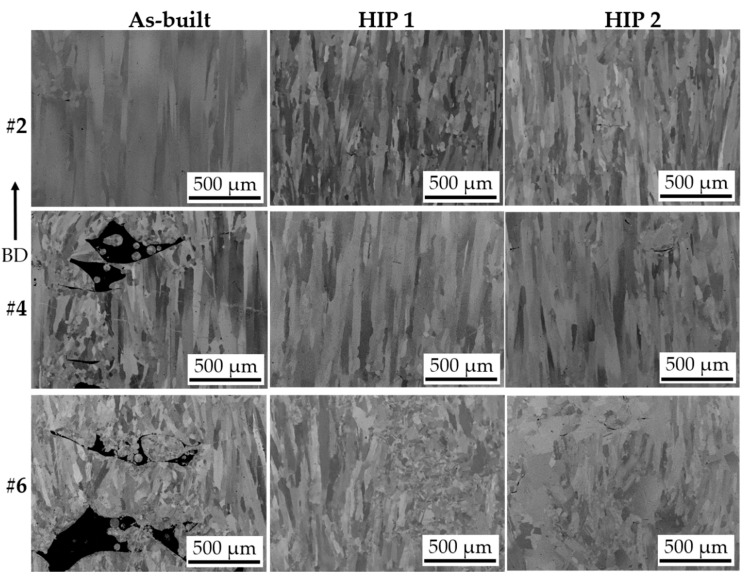
SEM micrographs showing defects in samples #2 (LS = 125 µm), #4 (LS = 225 µm), and #6 (LS = 325 µm) in as-built and HIP’ed conditions. The arrow indicates the build direction.

**Figure 4:**
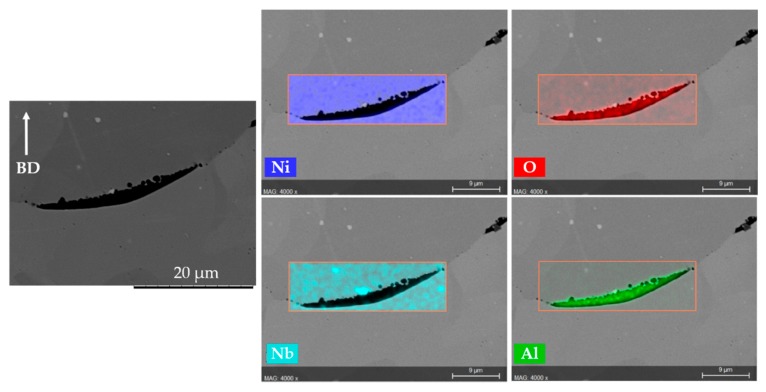
Representative EDS analysis showing inclusions typically associated with many remnant defects in sample #4 (LS = 225 µm) after HIP1. The arrow indicates the build direction.

Samples with distinct defect contents, i.e., AB#2, AB#4, and AB#6 were subjected to two HIPing treatments to investigate the efficacy of defect closure. The defect contents in as-built samples AB#2, AB#4, and AB#6 (corresponding to LS = 125, 225, and 325 µm, respectively) were 0.5%, 2.6%, and 17%, respectively. After both the HIPing treatments, the defect content in all samples was significantly reduced, regardless of the original extent, as summarized in Table 4. Moreover, the difference in the extent of defect closure accomplished at 1120 °C (HIP1) and 1185 °C (HIP2) was not significant. The considerable densification of samples achieved by HIPing is also visualized from the microstructures in Figure 3. Furthermore, the small amount of remnant defects in all HIP’ed samples discussed were found to be typically associated with the presence of Al-rich oxide, which can inhibit complete defect closure. This is clearly evident from the representative EDS results given in Figure 4. It is worth mentioning that Al-rich oxides are thermodynamically very stable and not removed by HIPing, as previously observed in HIP’ed EBM Alloy 718 [24].

It is pertinent to note that concerns regarding thermally induced porosity (TIP), i.e., regrowth of apparently “closed” defects during subsequent heat treatment, have been reported [25,26]. Therefore, the samples HIP1#2, #4, and #6 were also subjected to heat treatment at 1180° C/4 h under vacuum, as previously studied by Benn et al. [26]. The results showed no significant difference in defect content before and after the heat treatment. Therefore, the TIP phenomenon was not discernible in the present HIP’ed EBM built Alloy 718 samples, at least to a statistically discernible limit, thereby indicating the efficacy of defect closure achieved by HIPing. It is worth mentioning that TIP plausibly did not happen in the present case because of insignificant gas entrapment in the defects owing to the vacuum conditions employed during EBM processing [27]. This is in contrast to LPBF processing, during which there have been concerns related to process gas infiltration because the builds are carried out in an inert atmosphere, typically using argon [28].

Such extensive and efficient reduction in the defect content by orders of magnitude (as evident from Table 4 and Figure 3) *prima facie* provides an opportunity to also potentially increase productivity—at least to some reasonable extent—during EBM processing. It is worth mentioning that the melting step typically comprises ~35% (the exact value depends on the build design [22]) of the layer heating time (preheat + melt + post-heat), which corresponds to 15% of the total layer processing time (also including lowering of build platform + powder raking) spent during the EBM process. Therefore, decreasing the melting time (by increasing the LS, as done in this study) would also have a corresponding effect on the overall EBM processing time.

Consistent with the idea of taking a holistic approach to optimize the processing chain for EBM-built Alloy 718 components, the present study also sought to evaluate the role of shortened solutionizing and aging treatment (mentioned earlier) following HIPing. For this purpose, the samples HIP1#2, HIP1#4, and HIP1#6 were first subjected to a standard solution treatment followed by a shorter aging treatment (Table 3) in comparison to ASTM F3055 [19] specification, as previously suggested in a recent study carried out in the authors’ group [20]. The shortened aging treatment refers to a (740 °C/4 h cool at 55 °C/h to 635 °C, hold at 635 °C/1 h/AC to RT) cycle compared to the conventional (740 °C/8 h cool at 55 °C/h to 635 °C, hold at 635 °C/8 h/AC to RT) cycle suggested in the ASTM F3055 [19] specification.

**Figure 5:**
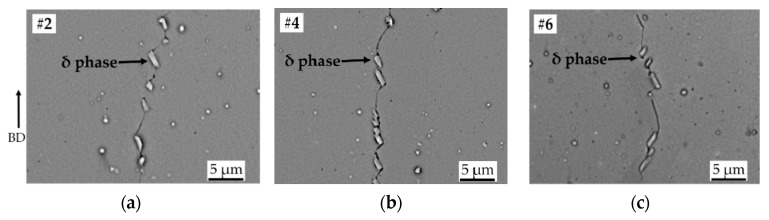
SEM micrographs of HIP1 + STA samples (**a**) LS = 125 µm, (**b**) LS = 225 µm, and (**c**) LS = 325 µm showing similar distribution of the δ phase. The arrow indicates the build direction.

**Figure 6:**
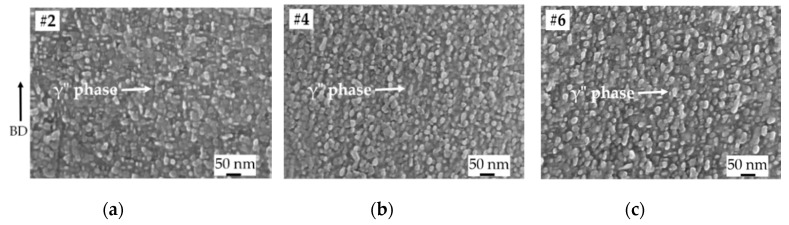
SEM micrographs of HIP1 + STA samples (**a**) LS = 125 µm, (**b**) LS = 225 µm, and (**c**) LS = 325 µm showing similar distribution of the γ″ phase. The arrow indicates the build direction.

Since the solution treatment was carried out at 954 °C, all the HIP1 + STA samples #2, #4, and #6 (corresponding to LS = 125, 225, and 325 µm, respectively) exhibited a virtually similar distribution of grain boundary δ phase, as shown in Figure 5a–c. This is in accordance with the reported time–temperature-transformation diagram of Alloy 718 [29], and has also been previously observed in solution-treated EBM Alloy 718 that was not subjected to prior HIPing [10]. The effect of aging treatment can be readily seen from the similar distribution of γ″ phase as shown in Figure 6a–c. These microstructural similarities in the three samples (HIP1 + STA #2, #4, #6) were also reflected in the microhardness results, as described below.

**Figure 7:**
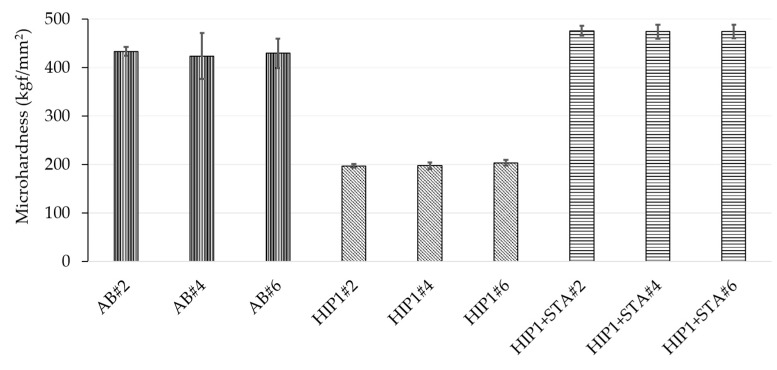
Microhardness values of the as-built and post-treated samples #2 (LS = 125 µm), #4 (LS = 225 µm), #6 (LS = 325 µm).

The average microhardness values of the as-built and post-treated samples, determined as previously described in the experimental procedure, are shown in Figure 7. It can be seen that, although the defect contents in the as-built samples were vastly different, they exhibit similar hardness values of ~430 kgf/mm^2^. This can be explained by the similarity in local melting conditions for the three as-built specimens, as the electron beam parameters were unchanged, and only the line spacing was varied. This contrasts a previous reported study by Lee et al. [23], where hardness was observed to vary (405–450 kgf/mm^2^) with defect content (4.5%–0.5%) of the EBM Alloy 718 samples. In the study by Lee et al. the local melting conditions were varied between the different specimens by changing the focus offset, thereby influencing energy density. The HIP1 treatment caused a reduction in the hardness of all the samples to nearly identical values of ~200 kgf/mm^2^. This is consistent with the previous observations on EBM and LPBF Alloy 718 [30,31]. After HIP1 + STA, all the samples exhibited similar hardness values (~475 kgf/mm^2^), which were higher than in the as-built condition. This value is close to the reported peak hardness (~490 kgf/mm^2^) of Alloy 718 [10]. Previous studies on EBM Alloy 718 have also reported an increase in hardness in the as-built condition from 410 kgf/mm^2^ to 470 kgf/mm^2^ after aging [32,33,34]. Thus, the foregoing results amply reveal that, regardless of the vastly different defect content in the as-built condition, the final post-treated condition exhibits a very similar density (Figure 3), phase constitution in terms of δ phase and γ″ phase distribution (Figure 5 and Figure 6), as well as microhardness values (Figure 7).

## 4. Conclusions

The present study on EBM Alloy 718 intentionally built with varied defect contents (by increasing hatch line spacing) clearly shows that regardless of the quantum of defects in the as-built condition, after HIPing, the material exhibits a nearly completely densified structure. The results are suggestive of the possibility of appropriate thermal post-treatments nullifying the influence of defects incorporated during EBM processing, thereby reducing elaborate process optimization efforts. A spin-off prospect of these findings can also be an opportunity to EBM print parts somewhat faster compromising defect content, if the production protocol is to involve an ensuing HIPing step in particular. In addition, subsequent solution treatment and ‘shortened’ aging compared to the conventional long aging yields a phase constitution and hardness that are virtually identical despite a vast difference in as-built defect contents. The resultant hardness was close to the peak hardness of Alloy 718. The above demonstrates the ability of thermal post-treatments involving HIPing, solutionizing and short aging treatments to render the defect content in as-built EBM Alloy 718 irrelevant. Such optimization of the build process and post-treatment together can have significant implications on industrialization of EBM technique.

## Figures and Tables

**Table 1:** EBM process parameters.

Sample Nomenclature	Hatch Line Spacing	Relative Melting Time Per Layer ^2^	Relative Melted Area Per Unit Time ^2^
	(LS, µm)		
#1	75	167%	0.6
#2 ^1^	125 *	100%	1 ^1^
#3	175	71%	1.4
#4	225	56%	1.8
#5	275	45%	2.2
#6	325	38%	2.6

^1^ Arcam recommended setting. ^2^ Values normalized with respect to the Arcam recommended setting.

**Table 2:** Process parameters in the Inco 4.2.205 theme for EBM Alloy 718.

Parameter	Value
Layer thickness (µm)	75
Acceleration voltage (kV)	60
Hatch scan rotation (°)	72
Pre-heat temperature (°C)	1025
Speed function	63
Focus offset (mA)	15
Max. beam current (mA)	18

**Table 3:** Details of post-treatments.

Designation	Cycle
HIP1	1120 °C/100 MPa/4 h/RC
HIP2	1185 °C/100 MPa/4 h/RC
HIP1 + STA	HIP1: 1120°C/100 MPa/4 h/RC +STA: 954 °C/1 h/WC to RT, 740° C/4 h/cool at 55 °C/h to 635 °C, hold at 635 °C/1 h/AC to RT

Note: The abbreviations RC, WC, AC, and RT denote rapid cooling, water cooling, air cooling, and room temperature, respectively.

**Table 4:** Defect content (vol.%) in various samples in as-built and HIP’ed conditions.

Sample	Condition		
	As-built	HIP1	HIP2
#2 (LS = 125 µm)	0.5 ± 0.3	0.1	0.1
#4 (LS = 225 µm)	2.6 ± 4.1	0.2 ± 0.1	0.3 ± 0.2
#6 (LS = 325 µm)	16.9 ± 11.3	0.2 ± 0.1	0.2 ± 0.1
